# Exploring the Dynamic Interplay of Deleterious Variants on the RAF1–RAP1A Binding in Cancer: Conformational Analysis, Binding Free Energy, and Essential Dynamics

**DOI:** 10.1002/prot.26759

**Published:** 2024-11-05

**Authors:** Abbas Khan, Syed Shujait Ali, Muhammad Ammar Zahid, Shahenda Salah Abdelsalam, Noorah Albekairi, Raed M. Al‐Zoubi, Mohanad Shkoor, Dong‐Qing Wei, Abdelali Agouni

**Affiliations:** ^1^ Department of Pharmaceutical Sciences, College of Pharmacy, QU Health Qatar University Doha Qatar; ^2^ Center for Biotechnology and Microbiology University of Swat Swat Pakistan; ^3^ College of Pharmacy King Saud University Riyadh Saudi Arabia; ^4^ Surgical Research Section, Department of Surgery Hamad Medical Corporation Doha Qatar; ^5^ Department of Biomedical Sciences, College of Health Sciences, QU Health Qatar University Doha Qatar; ^6^ Department of Chemistry Jordan University of Science and Technology Irbid Jordan; ^7^ Department of Chemistry, College of Arts and Science Qatar University Doha Qatar; ^8^ Department of Bioinformatics and Biostatistics, School of Life Sciences and Biotechnology Shanghai Jiao Tong University Shanghai China

**Keywords:** binding free energy, cancer, docking, MAPK, mutations, RAF1, simulation

## Abstract

The RAF1–RAP1A interaction activates the MAPK/ERK pathway which is very crucial in the carcinogenesis process. This protein complex influences tumor formation, proliferation, and metastasis. Understanding aberrant interactions driven by clinical mutations is vital for targeted therapies. Hence, the current study focuses on the screening of clinically reported substitutions in the *RAF1* and *RAP1A* genes using predictive algorithms integrated with all‐atoms simulation, essential dynamics, and binding free energy methods. Survival analysis results revealed a strong association between RAF1 and RAP1A expression levels and diminished survival rates in cancer patients across different cancer types. Integrated machine learning algorithms showed that among the 134 mutations reported for these 2 proteins, only 13 and 35 were classified as deleterious mutations in *RAF1* and *RAP1P*, respectively. Moreover, one mutation in *RAF1* reported elevated levels of binding between RAF1 and RAP1P while in *RAP1A*, 7 mutations were reported to increase the binding affinity. The high‐binding mutations, P34Q and V60F, were subjected to protein–protein coupling which confirmed the increase in the binding affinity. Wild‐type and mutant RAF1–RAP1P bound complexes were subjected to molecular simulation investigation, revealing enhanced structural stability, increased compactness, and stabilized residue fluctuations of the mutant systems in contrast to the wild‐type. In addition, hydrogen bonding analysis revealed a variation in the binding paradigm which further underscores the impact of these substitutions on the coupling of RAF1 and RAP1A. Principal component analysis (PCA) and free energy landscape (FEL) evaluation further determined dynamical variations in the wild‐type and mutant complexes. Finally, the Gibbs free energy for each complex was estimated and found to be −71.94 ± 0.38 kcal/mol for the wild‐type, −95.57 ± 0.37 kcal/mol for the V60F, and −85.76 ± 0.72 kcal/mol for P34Q complex. These findings confirm the effect of these variants on increasing the binding affinity of RAF1 to RAP1P. These mutations can therefore be targeted for cancer therapy to modulate the activity of the MAPK/ERK signaling pathway.

## 1 Introduction

Cancer is a major threat to public health globally both in developed and developing countries. The number of deaths linked to cancer is on the rise worldwide with every passing day and an expected increase of about 45% proliferation of cancer incidences has been predicted between 2010 and 2030. Among cancer types, pancreas, lung, and liver cancers have the highest contribution to cancer mortality [1, 2].

Cellular signaling pathways play a dominant and vital role in vital cellular processes, such as differentiation, proliferation, and survival. Dysregulation and alterations in these pathways lead to tumor formation, proliferation, and metastasis; which is a hallmark of cancer [3]. In cancer initiation, spread, and metastasis numerous signaling cascades have been reported, however, the Mitogen‐Activated Protein Kinase (MAPK)–Extracellular Signal‐Regulated Kinase (ERK) pathway has a central role in oncogenic transformation [4].

The role of RAF1 (Raf‐1 Proto‐Oncogene, Serine/Threonine Kinase) is a promising therapeutic target because it acts as a mediator, phosphorylating downstream targets such as MEK and ERK and passing extracellular signals to intracellular effectors [5]. In cancer patients, abnormal and aberrant RAF1 activation is associated with tumor growth and the development of therapy resistance. RAP1A (Ras‐Related Protein Rap‐1A) operates as an important regulator for cell invasion, migration, and adhesion in addition to promoting tumor metastasis [6]. RAP1A switches between GDP or GTP‐bound states and acts as a molecular switch that regulates the cellular activity of downstream effectors. The interaction of RAF1 with RAP1A and its signaling cascade has an essential role in the regulation of cellular proliferation and migration, and facilitates cellular responses including restructuring the cytoskeletal and regulating the gene expression levels [7]. The recent accumulation of vast genomic data identified a wide range of genetic variants, such as single‐nucleotide polymorphisms (SNPs) inside key signaling proteins. These mutations have a considerable impact on protein function and cellular activity [8]. However, the essential functional implications of *RAP1A* SNPs and their role in malignancies like cancer are not fully understood.

In this study, we wanted to know how RAP1A SNPs affected its interaction with RAF1 and their role in cancer susceptibility and development. We employed computational modeling, structural analysis, and functional experiments to investigate the effects of nonsynonymous *RAP1A* mutations on protein–protein interactions (PPI) and downstream signaling. In addition, we examined clinical data from cancer patient cohorts to assess the link between disruptive *RAP1A* SNPs and patient outcomes. Our research looks at the molecular mechanisms behind cancer's deregulation of the RAF1–RAP1A signaling axis using a multidisciplinary approach that combines bioinformatics, structural biology, and computational biology. Understanding the interaction of genetic variations and PPIs in cancer development holds promise for discovering prognostic markers and therapeutic targets, paving the way for customized oncology treatments. As a result, we evaluated the effect of recently found RAP1A protein mutations on RAP1A's capacity to bind to RAF1. To examine the interaction paradigm and decipher the mutations‐driven dynamic effect we molecular docking, protein–protein, and all‐atoms simulations to identify the binding network between RAP1A and RAF1 and docking results in a dynamic environment. The current study is unique in that it is the first to conduct a wide‐ranging computer simulation of these complexes, that will help in elucidating the subsequent impact of the shortlisted substitutions and can be used for precision therapeutics development.

## 2 Methods

### 2.1 Survival Analysis and RAF1–RAP1A Complex Retrieval

**FIGURE 1:**
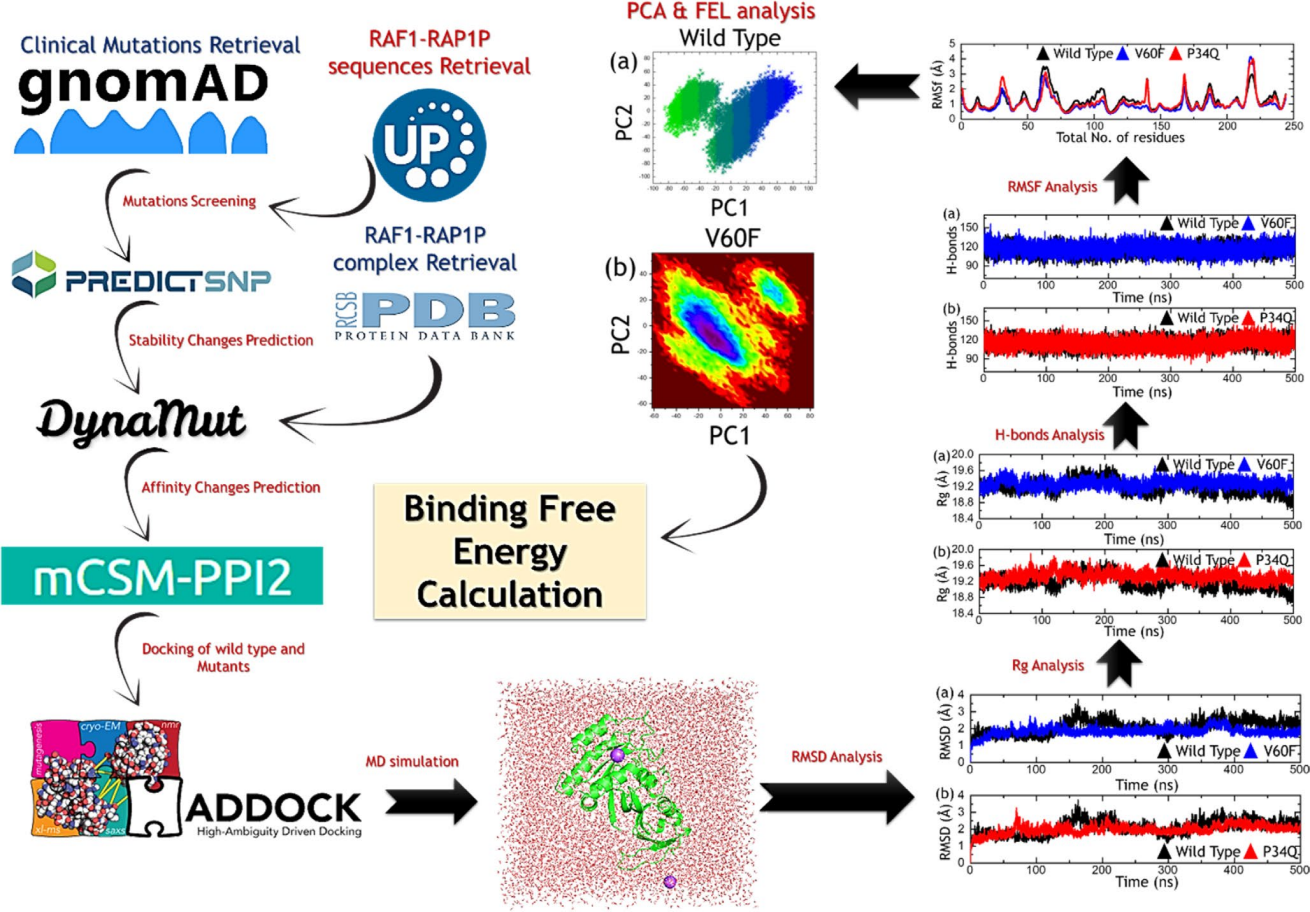
Systematic workflow of the study.

The survival and expression of individual genes against cancer with a possible effect on RAF1 and RAP1A were checked on different algorithms such as the Kaplan–Meier plotter (https://kmplot.com/analysis/) [9], and the interaction gene expression profiling database, that is, GEPIA2 were assessed (http://gepia2.cancer‐pku.cn/#survival) [10]. This tool is useful in analyzing clinical trials data as well as observational data linked with an event. GEPIA2 containing 9736 tumors and 8587 normal samples is a web‐based tool utilized for RNA sequencing expression data analysis obtained from the TCGA and GTEx projects [11, 12]. The data of the human *RAP1A* gene was submitted to the gnomAD database (https://gnomad.broadinstitute.org/) to obtain experimentally reported SNPs of this gene [13]. The 3D structural coordinates of RAF1 and RAP1A were collected from the Protein Data Bank (http://www.rcsb.org/) [14]. The complete steps and workflow of this study are shown in Figure 1.

### 2.2 Mapping the Structurally and Functionally Significant nsSNPs

Functionally, the impact of any substitution in a protein structure driven by non‐synonymous SNPs (nsSNPs) can be determined by using various algorithms such as PredictSNP [15], MAPP [16], PhD‐SNP [17], PolyPhen‐2 [18], PANTHER [19], and SIFT [20] that are deployed online for the public use. The detrimental nsSNPs predicted by the collective approach employing the aforementioned algorithms were shortlisted. The PredictSNP server integrated different computational algorithms (https://loschmidt.chemi.muni.cz/predictsnp1/) and combines data from experimental annotations in the pre‐deployed databases to predict the impact of nsSNPs. Furthermore, MAPP web tool accessible at (http://mendel.stanford.edu/SidowLab/downloads/MAPP/) was used for the functional impact prediction. The disease‐causing nsSNPs were obtained from the list by using PhD‐SNP (https://snps.biofold.org/phd‐snp/phd‐snp.html) and PolyPhen‐2 (http://genetics.bwh.harvest.edu/pp2) which uses a criterion of 0 to 1 which provides a numerical score from 0 to 1, with higher scores are the indications functional variations associated with an amino acid change. Similarly, the SIFT (Sorting Intolerant from Tolerant) (http://sift.bii.a‐star.edu.sg) is also helpful in producing the results that cause functional variants due to a mutation in a particular protein.

### 2.3 Exploring the Structural Stability Impact of RAP1A Mutations

The change in flexibility and stability of protein structure due to highly deleterious mutations was checked on the DynaMut2 (https://biosig.lab.uq.edu.au/dynamut/) web server [21]. Mutations deemed as highly damaging were subjected to structural stability assessment. The results of the DynaMut were used to identify the best mutations which were subsequently used for affinity variations between RAF1 and RAP1A using mCSM‐PPI2 (https://biosig.lab.uq.edu.au/mcsm_ppi2/) [22]. Utilizing a machine learning technique, this server employs graph‐based structural signatures, mCSM, offering a precise and scalable means to forecast mutations' function association and enhancing the understanding of mutation molecular mechanisms by exploring the inter‐residue non‐covalent interaction network. This is achieved through the integration of graph kernels, evolutionary insights, complex network metrics, and energetic considerations.

### 2.4 Variant Modeling and Docking of RAF1 With RAP1A

RAF1–RAP1A (PDB ID: 1C1Y) co‐crystal structure was downloaded from the PDB and was subjected to an energy minimization step carried on the Chimera software [23]. With the help of this software, highly detrimental and affinity‐increasing mutations in the RAF1–RAP1A complex were modeled. The superimposition of the wild‐type and mutant RAF1–RAP1A structures was performed for RMSD differences calculation. Molecular docking is used for the binding of molecules with specific 3D orientation and was performed by using the HADDOCK server (https://wenmr.science.uu.nl/haddock2.4/) to assess the mutations' impact on the binding affinity of RAF1 and RAP1A [24]. HADDOCK stands out from ab initio docking methodologies by incorporating data from known or predicted interfaces of different proteins into ambiguous interaction restraints (AIRs) to guide the docking procedure. Additionally, it assists the description of specific unambiguous distance restraints such as MS cross‐links and accommodates various other experimental data types such as NMR residual dipolar couplings, pseudo contact shifts, and cryo‐EM maps. HADDOCK is versatile and capable of addressing a wide range of modeling challenges, such as biological macromolecules, including assemblies with multiple bodies (*N* > 2).

### 2.5 Molecular Dynamics Simulation

Mutations that were reported to destabilize the protein structures as well as significantly affect the binding were used for further validations to decipher variations in the dynamic properties of each protein by using molecular simulations with the AMBER23 tool using the ff19SB as suggested by previous studies [11, 12]. For the solvation process, an OPC water box was used (with a cut‐off of 10.0 Å), whereas neutralization was achieved by the addition of sodium ions. Complexes were passed through a two‐step minimization process with the help of reported protocols. RAP1A protein was subjected to 300 ns simulation and 500 ns simulation was performed for the complexes using constant temperature and pressure achieved Langevin thermostat set at 300 K and 1 atm, respectively. Computation of long‐range interactions was ascertained by the particle mesh Ewald (PME) algorithm with a cut‐off distance of 10 Å, and the SHAKE algorithm was used for covalent bonds involving hydrogen [25, 26, 27]. We executed a 50 ns equilibrium for each complex. The GPU‐accelerated PMEMD‐CUDA was utilized to process the simulations. For the calculations of structural stability, we used root mean square deviation (RMSD) a well‐known parameter for calculating the stability of a protein in a dynamic condition, RMSF or root mean square fluctuation (RMSF) to index the flexibility level of different residues and hydrogen bonding analysis over the simulation time, while for the protein size determination during the simulation, the radius of gyration (*R*_*g*_) was computed [28, 29].

### 2.6 Binding Free Energy Calculation

MM/GBSA approach is very useful for the determination of the binding free energy (BFE) for wild‐type and mutant complexes. Previously this approach has provided reliable results related to BFE for various biological systems [30, 31, 32, 33]. To calculate the BFE we executed the MMPBSA.py script [34] using the following equation.

(1) $$\Delta G\left(\text{bind}\right)=\Delta G\left(\text{complex}\right)-\left[\Delta G\left(\text{receptor}\right)+\Delta G\left(\text{ligand}\right)\right]$$

where Δ*G*_bind_ is the total binding energy, Δ*G*_complex_ is the binding energy protein–ligand complex, Δ*G*_receptor_ is the binding energy for receptor only, and Δ*G*_ligand_ is the binding energy for ligand only.

Each component was then individually characterized and calculated to provide further insights into each contributing factor.

(2) $$\;G=G_{\text{bond}}+G_{\mathrm{ele}}+G_{\mathrm{vdW}}+G_{\mathrm{pol}}+{\mathrm{Gn}}_{\mathrm{pol}}$$

where Δ*G*_bond_ signifies the binding, Δ*G*_ele_ shows the electrostatic contribution, Δ*G*_vdw_ shows the Van Der Waals contribution, Δ*G*_pol_ shows the polar contribution, and Δ*Gn*_pol_ shows the non‐polar contribution.

### 2.7 Principal Component and Free Energy Landscape Analysis

The fluctuation in the protein structure was captured by principal component analysis (PCA). Cα coordinates were used for computing a covariance matrix by using the CPPTRAJ package, whereas eigenvectors and eigenvalues were obtained by diagonalization of this matrix. The eigenvectors are used for motion directions and eigenvalues are used for the extent of mean square fluctuation. The protein's motion was traced by PC1 and PC2 [35, 36, 37]. A mathematical representation given in Equation (3) was used to estimate the covariance matrix (*C*) from a set of *n*‐dimensional vectors (*x*_*i*_):

(3) $$C=\frac{1}{N}\times \sum \left(i=1\;\text{to}\;N\right)\left[\;(x_{i}-\mu \right)\times {\left(x_{i}-\mu \right)}^{T}]$$

where, *N* stands for total vectors, *μ* signifies a vector, while *T* defines the transpose operation.

Similarly to eigenvectors (*V*) and eigenvalues (*λ*) of the covariance matrix *C* the equation given (4) can be used.

(4) C×V=λ×V

where *V* is the eigenvector matrix and *λ* is the diagonal matrix of eigenvalues.

The principal components of the system are denoted by the eigenvectors with the highest corresponding eigenvalues. In the FEL, stable low‐energy states are represented by deep valleys on the plot, while intermediate states are depicted by the boundaries between these valleys.

## 3 Results and Discussion

### 3.1 Survival and Expression Analysis of RAF1 and RAP1A in Normal and Cancerous Cells

**FIGURE 2:**
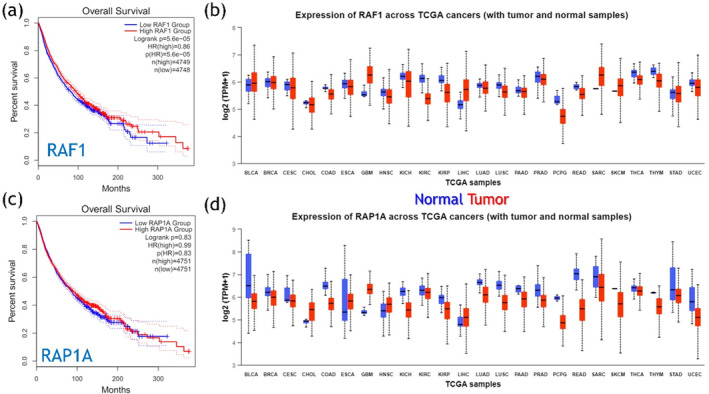
The part figures (a) and (b) show survival and expression plots for *RAF1*, while (c) and (d) show the survival and expression plots for *RAP1A*. The figures were obtained from GEPIA2 and ProteinATLAS.

The Kaplan–Meier (KM) Plotter determines the correlation among genes and protein expression (such as Protein, miRNA, and mRNA) and survival results in a bulky dataset encompassing over 30 000 samples across 21 different types of tumors. Its main objective is to facilitate the finding and validation of biomarkers capable of predicting survival outcomes through meta‐analysis in cancer patients. To examine the relationship between RAF1 and RAP1A expression intensities in tumor tissues and cancer patient prognoses, we explored survival analyses across the pan‐cancers. Utilizing three distinct databases, and then evaluated the survival data. The results of survival analyses from the KM‐Plotter indicated that altered expressions of these genes are linked to reduced survival rates across different cancer types. Similarly, analyses using the GEPIA2 database revealed expression variations of *RAF1* and *RAP1A* among the cancer types assessed. Our analysis highlights a strong association between RAF1 and RAP1A expression levels and diminished survival rates in cancer patients across the different cancer types (Figure 2a–d).

### 3.2 Screening of Deleterious Clinical Substitutions in RAF1

We retrieved a total of 62 clinical variants of RAF1 protein among which only 13 were classified as deleterious by a consensus outcome of multiple algorithms. These 13 substitutions include V60F with the PredictSNP score of 0.869, R111C (0.506), R59H (0.719), D129V (0.719), N56H (0.506), R59C (0.655), F61L (0.549), P63L (0.869), R67T (0.719), R73Q (0.605), G75R (0.756), V98A (0.607), and D117V (0.719). These mutations were further explored for their potential impact on the binding of RAF1–RAP1A complex and aberrant function. The selected mutations are summarized in Table 1.

**TABLE 1:** List of deleterious mutations predicted through various machine learning algorithms for the RAF1 protein.

Mutation	Consensus outcome	Predict SNP score	MAPP score	PhD‐SNP	Polyphen‐1	Polyphen2	SIFT	SNAP	Panther
V60F	Deleterious	0.869	0.589	0.745	0.650	0.793	0.805	0.000	0.000
R111C	Deleterious	0.506	0.783	0.745	0.562	0.737	0.720	0.000	0.000
R59H	Deleterious	0.719	0.766	0.858	0.594	0.601	0.793	0.848	0.567
D129V	Deleterious	0.719	0.678	0.589	0.594	0.407	0.793	0.720	0.567
N56H	Deleterious	0.506	0.760	0.783	0.745	0.431	0.452	0.622	0.557
R59C	Deleterious	0.655	0.766	0.875	0.594	0.474	0.528	0.805	0.548
F61L	Deleterious	0.549	0.766	0.578	0.594	0.628	0.528	0.622	0.557
P63L	Deleterious	0.869	0.484	0.875	0.745	0.811	0.793	0.805	0.000
R67T	Deleterious	0.719	0.750	0.858	0.745	0.601	0.793	0.720	0.000
R73Q	Deleterious	0.605	0.790	0.773	0.594	0.647	0.608	0.622	0.000
G75R	Deleterious	0.756	0.705	0.858	0.745	0.811	0.793	0.720	0.000
V98A	Deleterious	0.607	0.653	0.817	0.669	0.675	0.793	0.805	0.000
D117V	Deleterious	0.719	0.705	0.858	0.745	0.601	0.793	0.556	0.000

### 3.3 Screening of Deleterious Clinical Substitutions in *RAP1A*

The mutational screening of RAP1A clinical substitutions revealed 35 mutations, among the 134 identified, as deleterious and led to functional variations. The top 10 deleterious mutations as per the PredictSNP scores include R2C (0.756), V7M (0.869), G12V (0.869), V14I (0.869), A18V (0.869), L19Q (0.869), Q22L (0.869), D33Y (0.869), P34Q (0.869), and P34L (0.869). These mutations affect the structure and function of RAP1A in a manifold compared with the others. The predicted 35 deleterious mutations are summarized in Table 2.

**TABLE 2:** List of deleterious mutations predicted through various machine learning algorithms for the RAP1A protein.

Mutation	Consensus outcome	Predict SNP score	MAPP score	PhD‐SNP score	PolyPhen‐1 score	PolyPhen‐2 score	SIFT score	SNAP score	Panther score
R2C	Deleterious	0.756	0.409	0.875	0.745	0.601	0.459	0.554	0.760
R2L	Deleterious	0.719	0.484	0.858	0.745	0.398	0.459	0.554	0.745
Y4H	Deleterious	0.506	0.735	0.552	0.745	0.811	0.430	0.665	0.743
V7M	Deleterious	0.869	0.571	0.817	0.745	0.811	0.793	0.622	0.745
S11P	Deleterious	0.719	0.409	0.885	0.745	0.675	0.430	0.584	0.766
G12V	Deleterious	0.869	0.560	0.875	0.745	0.811	0.793	0.805	0.745
V14I	Deleterious	0.869	0.633	0.817	0.745	0.601	0.430	0.556	0.874
A18V	Deleterious	0.869	0.573	0.817	0.745	0.811	0.459	0.622	0.874
L19Q	Deleterious	0.869	0.589	0.676	0.745	0.811	0.793	0.720	0.874
Q22K	Deleterious	0.605	0.731	0.885	0.594	0.431	0.459	0.500	0.744
Q22L	Deleterious	0.869	0.858	0.875	0.745	0.593	0.793	0.556	0.745
D33Y	Deleterious	0.869	0.509	0.817	0.745	0.811	0.793	0.720	0.780
P34Q	Deleterious	0.869	0.858	0.773	0.745	0.811	0.793	0.720	0.780
P34L	Deleterious	0.869	0.857	0.608	0.745	0.811	0.793	0.622	0.842
T35A	Deleterious	0.869	0.920	0.817	0.745	0.453	0.430	0.556	0.842
T35M	Deleterious	0.869	0.857	0.858	0.745	0.811	0.793	0.720	0.842
S39P	Deleterious	0.756	0.621	0.733	0.745	0.601	0.528	0.500	0.842
S39Y	Deleterious	0.756	0.484	0.447	0.745	0.650	0.528	0.556	0.766
D47Y	Deleterious	0.869	0.621	0.875	0.745	0.675	0.793	0.622	0.874
C48W	Deleterious	0.607	0.560	0.773	0.745	0.610	0.793	0.554	0.743
C51R	Deleterious	0.869	0.857	0.875	0.745	0.431	0.459	0.622	0.760
L53P	Deleterious	0.869	0.807	0.875	0.745	0.593	0.528	0.622	0.874
E54K	Deleterious	0.549	0.571	0.608	0.669	0.644	0.430	0.720	0.719
T58I	Deleterious	0.869	0.765	0.773	0.745	0.811	0.793	0.720	0.766
A59V	Deleterious	0.869	0.657	0.733	0.745	0.811	0.793	0.720	0.780
D69Y	Deleterious	0.869	0.462	0.676	0.745	0.811	0.793	0.556	0.780
V81A	Deleterious	0.719	0.657	0.608	0.669	0.453	0.793	0.556	0.874
S83P	Deleterious	0.869	0.761	0.875	0.745	0.811	0.793	0.720	0.874
T85K	Deleterious	0.869	0.589	0.858	0.745	0.453	0.793	0.622	0.842
T89M	Deleterious	0.869	0.771	0.773	0.745	0.650	0.793	0.805	0.874
L96P	Deleterious	0.869	0.783	0.858	0.745	0.601	0.793	0.720	0.874
R97M	Deleterious	0.506	0.697	0.552	0.745	0.593	0.793	0.554	0.744
R102W	Deleterious	0.756	0.509	0.858	0.745	0.675	0.793	0.500	0.874
V114A	Deleterious	0.506	0.621	0.817	0.669	0.628	0.459	0.500	0.745
C118R	Deleterious	0.719	0.633	0.858	0.594	0.702	0.528	0.720	0.719
D119N	Deleterious	0.869	0.765	0.817	0.745	0.811	0.793	0.805	0.874
L120V	Deleterious	0.719	0.751	0.733	0.594	0.398	0.528	0.554	0.780
L120M	Deleterious	0.605	0.678	0.733	0.745	0.551	0.528	0.584	0.766
R124Q	Deleterious	0.869	0.484	0.885	0.594	0.811	0.793	0.720	0.842
W138C	Deleterious	0.869	0.409	0.589	0.745	0.398	0.793	0.720	0.745
C141R	Deleterious	0.869	0.877	0.773	0.745	0.431	0.793	0.848	0.874
L161M	Deleterious	0.756	0.589	0.676	0.745	0.431	0.793	0.584	0.842
R163G	Deleterious	0.605	0.588	0.447	0.594	0.683	0.430	0.556	0.842
R167M	Deleterious	0.549	0.789	0.589	0.745	0.503	0.459	0.500	0.745
C181R	Deleterious	0.756	0.766	0.773	0.745	0.453	0.793	0.554	0.567

### 3.4 Stability and Functional Variation Prediction Using Graph‐Based Signatures

We further determined the impact of stability changes and coupling of RAF1 with RAP1A using graph‐based signatures. Using DynaMut 2.0, the 13 deleterious mutations were subjected to stability outcome prediction. Among the 13 deleterious mutations, 9 mutations were reported to destabilize the protein while 4 mutations were reported to increase the stability of RAF1. On the other hand, using mCSM‐PPI2, 12 mutations were reported to decrease the binding of RAF1 with RAP1A while a single mutation, V60F, with an affinity change score of 0.051 was reported to increase the binding of RAF1 with RAP1A.

Hence, we speculated that these mutations could potentially perturb the cellular cascades and therefore contribute to the disease phenotype. However, these results are based on a single structure and its potential impact should be validated using a molecular dynamics (MD) simulation approach. The DynaMut and mCSM‐PPI2 results are summarized in Table 3.

**TABLE 3:** Prediction of stability changes and affinity variations using a graph‐based signature algorithm for the RAF1 protein.

Mutation	DynaMut prediction	Stability outcome	mCSM‐PPI score	Affinity outcome
R67T	−1.39	Destabilizing	−1.358	Decreasing
F61L	−1.65	Destabilizing	−0.755	Decreasing
D129V	0.63	Stabilizing	−0.258	Decreasing
R111C	0.8	Stabilizing	−0.459	Decreasing
R59C	−1.36	Destabilizing	−0.904	Decreasing
V98A	−1.78	Destabilizing	−0.401	Decreasing
P63L	−0.43	Destabilizing	−0.475	Decreasing
R59H	−1.22	Destabilizing	−0.522	Decreasing
V60F	−1.57	Destabilizing	0.051	Increasing
R73Q	−0.27	Destabilizing	−0.272	Decreasing
D117V	0.13	Stabilizing	−0.302	Decreasing
G75R	0.1	Stabilizing	−0.143	Decreasing

In the case of RAP1A, 12 mutations were classified as destabilizing while 23 mutations were reported to increase the stability. Among the 35 mutations in RAP1A, 7 mutations were reported to upsurge the binding affinity however 28 were classified to reduce the binding affinity. Among the affinity‐increasing mutations, A18V (0.056), R102W (0.059), D33Y (0.109), P34Q (0.230), G12V (0.077), D47Y (0.142), and D69Y (0.105) are involved while the rest were affinity decreasing. The mutation, P34Q, was selected for the subsequent analysis as it has shown the highest affinity change while being a destabilizing substitution. The DynaMut and mCSM‐PPI2 results for RAP1A are shown in Table 4. While analyzing these mutations, it can be seen that some mutations either stabilize or destabilize a protein. This could be due to several factors such as hydrophobicity, charge, size, and secondary structure propensity, along with the specific structural context of the mutation site, play crucial roles in determining the impact of mutations on protein stability. For instance, the mutation D129V is stabilizing, and is probably due to the introduction of a more hydrophobic amino acid that favors core packing. In contrast, R67T is likely to be destabilizing because this substitution tends to eliminate crucial hydrogen bonding or electrostatic interactions. The mutation L96P is destabilizing possibly due to the inclusion of proline, which has a propensity to disturb secondary structure formation, for example, alpha helices. In contrast, the same R111C mutation enhances the stability of the protein, probably due to additional disulfide bonds formed increasing the rigidity of the structure. Also, V60F induces a destabilization, perhaps owing to steric clashes in the protein core. Although these results are reported to affect the structure and function differently; however, these results are based on a single structure, and further validation through MD‐based data should be performed.

**TABLE 4:** Prediction of stability changes and affinity variations using a graph‐based signature algorithm for the RAP1A protein.

Mutations	DynaMut prediction	Stability outome	mCSM‐PPI score	Affinity outcome
L96P	−1.70	Destabilizing	−0.722	Decreasing
L120M	0.39	Stabilizing	−0.365	Decreasing
Q22K	0.03	Stabilizing	−0.649	Decreasing
A18V	−0.60	Destabilizing	0.056	Increasing
R102W	−0.33	Destabilizing	0.059	Increasing
C118R	−0.09	Destabilizing	−0.072	Decreasing
R124Q	−0.77	Destabilizing	−0.564	Decreasing
R2C	0.61	Stabilizing	−0.074	Decreasing
P34L	−0.47	Destabilizing	−0.33	Decreasing
D33Y	0.33	Stabilizing	0.109	Increasing
P34Q	0.08	Stabilizing	0.230	Increasing
T85K	−0.59	Destabilizing	−0.229	Decreasing
R2L	1.20	Stabilizing	−0.252	Decreasing
T35M	0.41	Stabilizing	−0.623	Decreasing
L19Q	−1.78	Destabilizing	−0.386	Decreasing
C48W	−0.30	Stabilizing	0.37	Increasing
R167M	−0.15	Destabilizing	−0.385	Decreasing
L161M	−2.06	Destabilizing	−0.279	Decreasing
S11P	−0.56	Stabilizing	−0.147	Decreasing
V81A	−2.01	Destabilizing	−0.597	Decreasing
V7M	−0.60	Destabilizing	−0.439	Decreasing
G12V	−1.36	Destabilizing	0.077	Increasing
V14I	−0.36	Destabilizing	−0.118	Decreasing
S83P	−0.84	Destabilizing	−0.832	Decreasing
V114A	−1.99	Destabilizing	−0.671	Decreasing
L53P	−1.68	Destabilizing	−0.955	Decreasing
Q22L	−0.45	Stabilizing	−0.301	Decreasing
R163G	−1.26	Destabilizing	−0.242	Decreasing
T58I	−0.52	Destabilizing	−0.152	Decreasing
T35A	−0.06	Destabilizing	−1.014	Decreasing
Y4H	−1.91	Destabilizing	−0.308	Decreasing
R97M	−1.27	Destabilizing	−0.379	Decreasing
D119N	−0.46	Destabilizing	−0.32	Decreasing
D47Y	0.34	Stabilizing	0.142	Increasing
D69Y	0.47	Stabilizing	0.105	Increasing
C141R	−0.44	Destabilizing	−0.085	Decreasing
S39Y	−1.23	Destabilizing	−0.651	Decreasing
E54K	−1.30	Destabilizing	−1.618	Decreasing
C51R	−1.36	Destabilizing	−0.004	Decreasing
T89M	−0.48	Destabilizing	−0.474	Decreasing
W138C	−1.88	Destabilizing	−0.593	Decreasing
S39P	−0.60	Destabilizing	−1.925	Decreasing
A59V	−0.89	Destabilizing	−0.043	Decreasing
L120V	−1.46	Destabilizing	−0.001	Decreasing

### 3.5 Dynamics‐Based Stability Investigation

**FIGURE 3:**
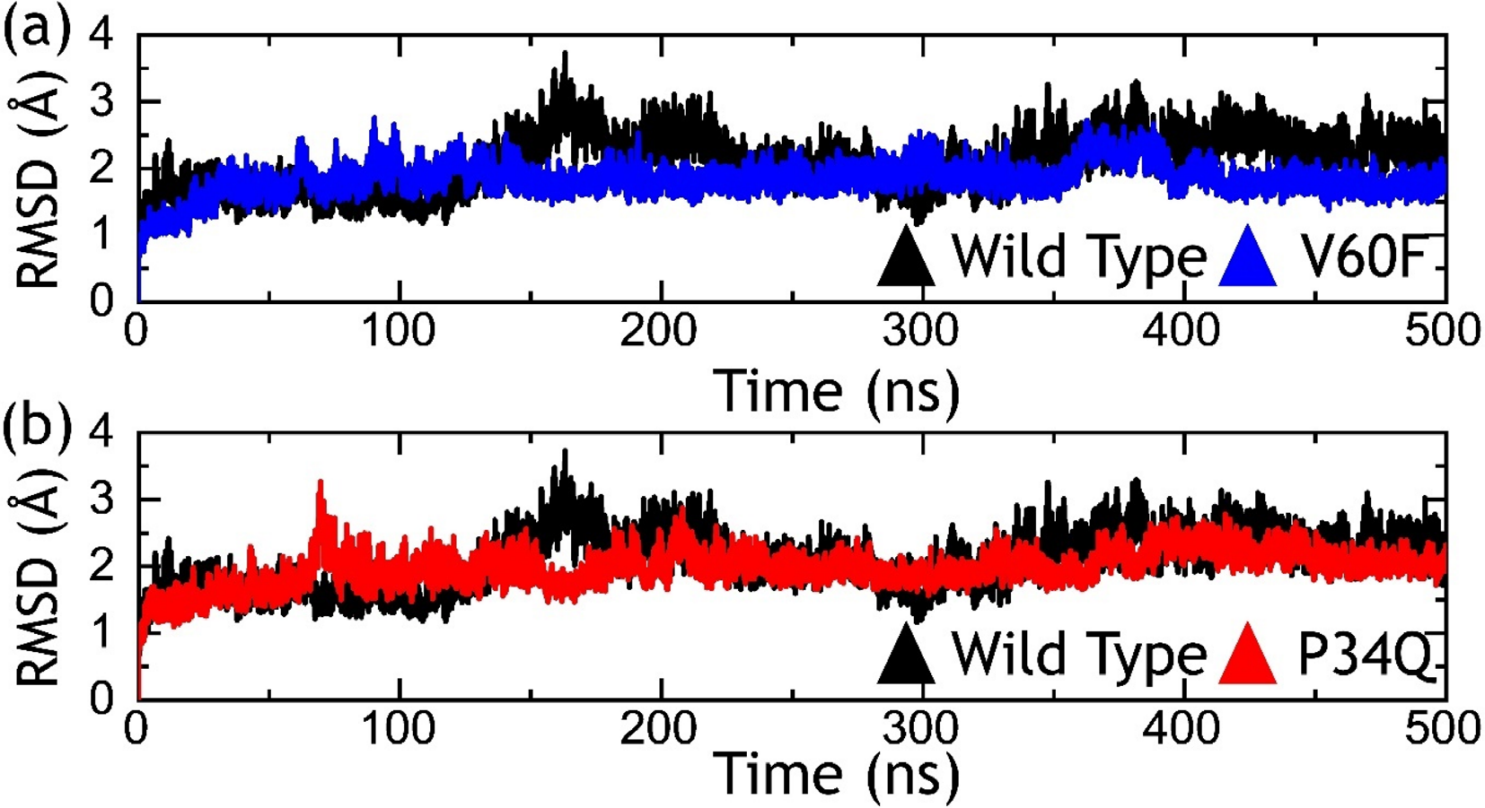
Dynamics stability investigation using RMSD metrics. The part figure (a) shows the RMSD graphs for the wild‐type and V60F systems, while (b) shows the RMSD graphs for the wild‐type and P34Q systems.

Determination of RMSD is a significant parameter for evaluating the structural stability and dynamics of a molecular system. It quantifies the average distance between corresponding atoms in different frames of a simulation compared with a reference structure. Calculation of RMSD provides an understanding of the conformational variations experienced by the macromolecules over time and consequently reports its flexibility, stability, and overall structural dynamics. In the context of macromolecular function, RMSD provides essential information regarding the structural motifs, active sites, and binding interfaces, vital for comprehending molecular interactions and guiding drug discovery efforts [33, 38, 39]. Considering the higher importance of RMSD metrics we computed RMSD by using the time‐dependent simulation trajectory for each complex. As shown in Figure 3a, the wild‐type reported a comparatively unstable behavior during the simulation. The wild‐type complex reported a higher RMSD at 180 ns and similar behavior was experienced at different time intervals during the simulation. The RMSD stabilized at 2.20 Å and maintained a uniform level after 400 ns. In contrast, the V60F mutation in the RAF1 (RAF1–RAP1A complex) had a comparatively lower RMSD. No significant structural perturbation was seen and the average RMSD 1.80 Å was reported. The mutation, V60F, consequently stabilizes the structure and thus causes functional variance. The RMSD for the RAF1–RAP1A V60F complex is shown in Figure 3a. On the other hand, the P34Q mutation reported a more similar behavior as the wild‐type although keeping an average RMSD lower than the wild‐type. The complex reported minor perturbation during the first 220 ns and then maintained a lower RMSD value until the end of the simulation. An average RMSD was reported to be 2.01 Å for the P34Q mutant system. The RMSD graph for the P34Q complex is shown in Figure 3b. The analysis of RMSD profiles for these systems, that is, RAF1–RAP1A complexes discloses distinct structural behaviors during the simulation and proposes that the V60F mutations particularly induce structural stability in a dynamic environment, consequently causing functional alterations. In sum, the RMSD analysis reveals how these mutations affect the structural dynamics of these complexes and offers insights into the functional implications of those changes.

### 3.6 Structural Compactness Analysis Through *R*_*g*_ Calculation

**FIGURE 4:**
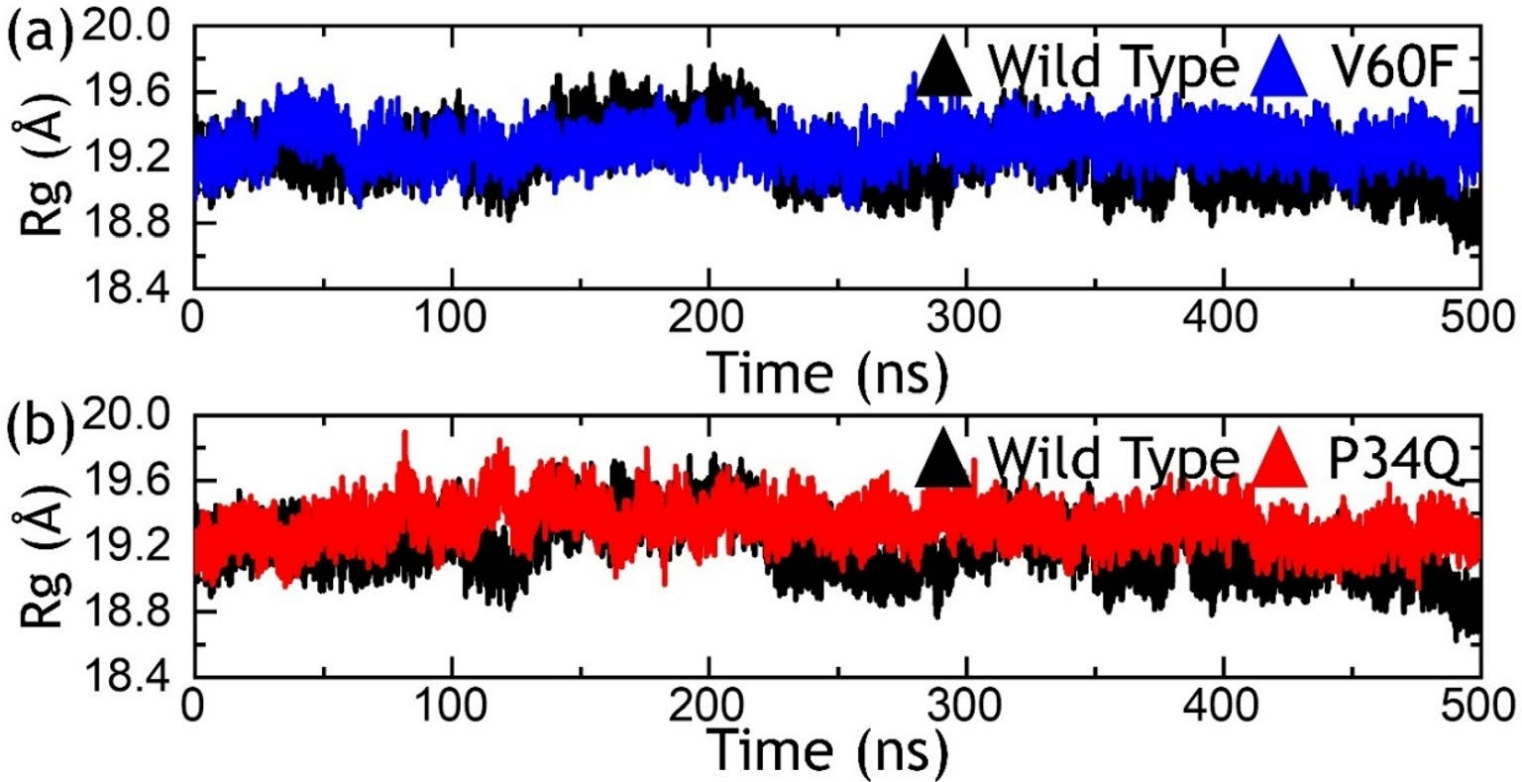
Structural compactness investigation using *R*_*g*_ metrics. The part figure (a) shows the *R*_*g*_ graphs for the wild‐type and V60F systems, while (b) shows the *R*_*g*_ graphs for the wild‐type and P34Q systems.

Measuring the distance from the center of mass to depict the protein's size can offer insights into its functional implications and variations in conformational dynamics. This method facilitates the characterization of novel interactions and the binding or dissociation of specific partners. Therefore, to assess the impact of the mentioned mutations on the size of these proteins during simulation, we conducted *R*_*g*_ calculations over time, as illustrated in Figure 4a,b. The *R*_*g*_ pattern for the wild‐type started to increase gradually and reached the maximum at 200 ns; however, then continued to decrease gradually and a more compact complex was obtained at the end of the simulation. In contrast, the V60F mutation maintained a similar level throughout the simulation and therefore demonstrated similar behavior as the RMSD. The *R*_*g*_ results for the wild‐type and V60F complexes are given in Figure 4a. On the other hand, the P34Q also reported a uniform *R*_*g*_ pattern with no significant structural perturbation throughout the simulation. An average *R*_*g*_ for the P34Q was estimated to be 19.40 Å. The *R*_*g*_ graph for the P34Q is shown in Figure 4b. This indicates that these mutations cause variation in the protein dynamics and cause minimal unbinding events throughout the simulation.

### 3.7 Residues Flexibility Analysis

**FIGURE 5:**
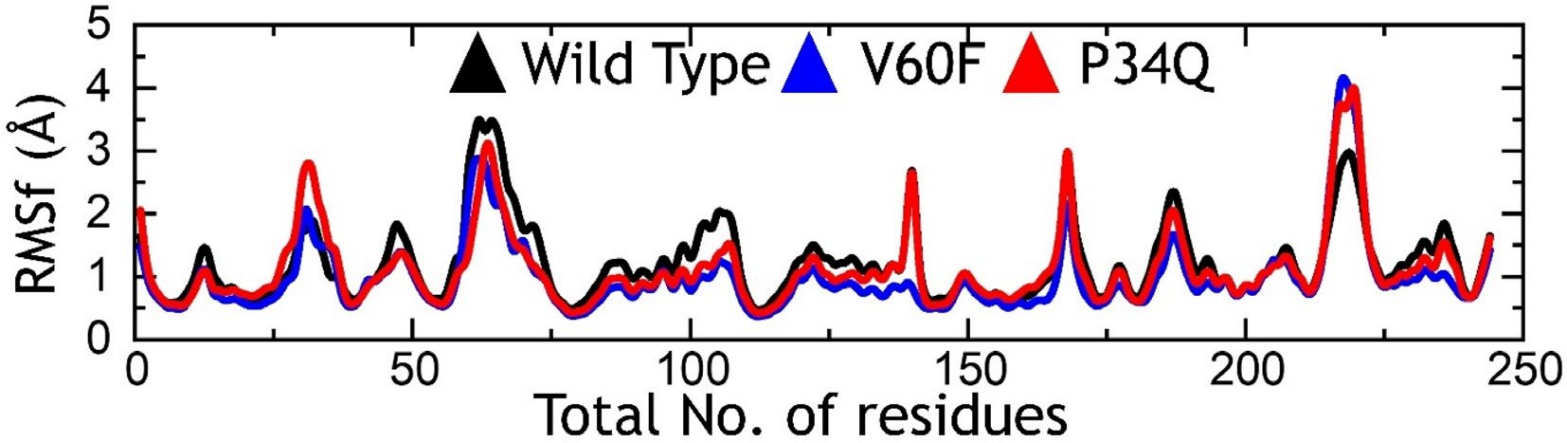
Residue's flexibility analysis of the wild‐type, V60F, and P34Q.

In the realm of MD simulations, the RMSF serves as a valuable tool for assessing the flexibility of distinct regions within a molecule and comparing it across various molecules. This metric aids in the identification of pivotal flexible regions, which may play a crucial role in ligand binding or protein–protein interactions. Moreover, RMSF as a method for calculating the flexibility holds significance as a to validate MD simulations which can be compared with the RMSF through experimental. Understanding the association between experimental and force field based flexibility values underscores the fidelity of the simulation in capturing the biomolecule's flexibility and dynamics. To assess the impact of mutations on the internal residue flexibility of the wild‐type and mutant proteins, RMSF calculations were conducted. All the complexes reported higher residue flexibility. The regions 25–35, 55–75, 165–175, 182–190, and 214–225 reported higher fluctuations while the others reported minimal fluctuations. The V60F mutation particularly reported minimal fluctuations and therefore demonstrated the stabilization of internal fluctuations upon the coupling. These results show the impact of these substitutions on the internal fluctuations in a dynamic environment and demonstrate the variations in the dynamic behavior. The RMSF graphs for the wild‐type, V60F, and P34Q are shown in Figure 5.

### 3.8 Hydrogen Bonding Analysis

**FIGURE 6:**
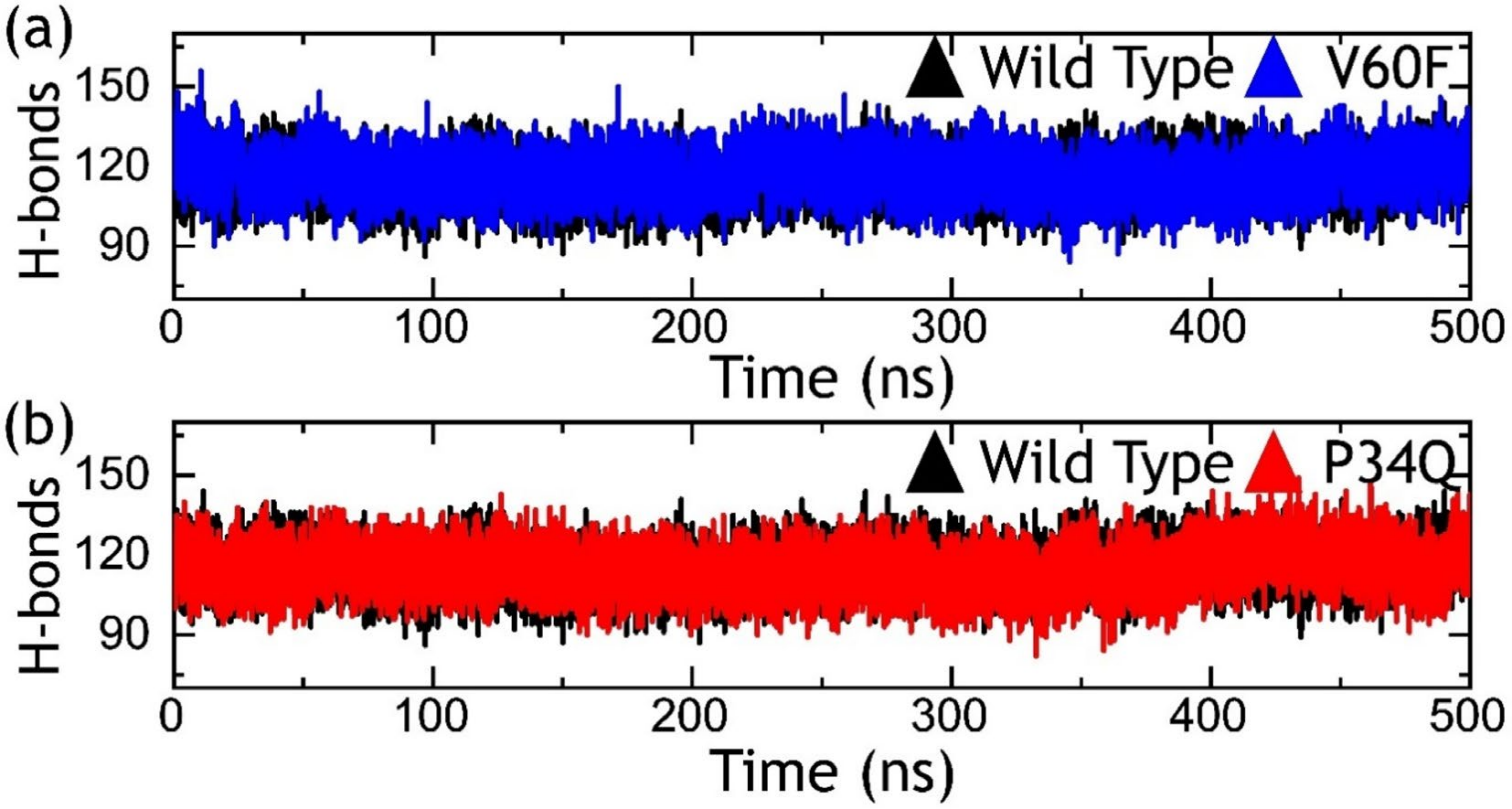
Hydrogen bonding analysis of the wild‐type and mutant. The part figure (a) shows the H‐bonds graphs for the wild‐type and V60F systems, while (b) shows the H‐bonds graphs for the wild‐type and P34Q systems.

Hydrogen bonds, exclusively within protein–protein contacts, play a crucial role in unraveling the mechanisms underlying various biological pathways, disease mechanism, and the effects of mutations on proteins binding in a molecular process. Recognizing the pivotal contribution of hydrogen bonding in numerous biological phenomena, we also conducted evaluations of hydrogen bonds within each trajectory throughout the simulation period. As shown in Figure 6a,b, the hydrogen bonding graph over the simulation time was calculated. In the case of the wild‐type 115 average number of hydrogen bonds while in the V60F the average hydrogen bonds were calculated to be 117. On the other hand, in the P34Q the average hydrogen bonds were estimated to be 118. This shows the differential hydrogen bonding paradigm in each complex due to these substitutions. We also calculated the hydrogen bonding in each complex using the equilibrated structure.

**FIGURE 7:**
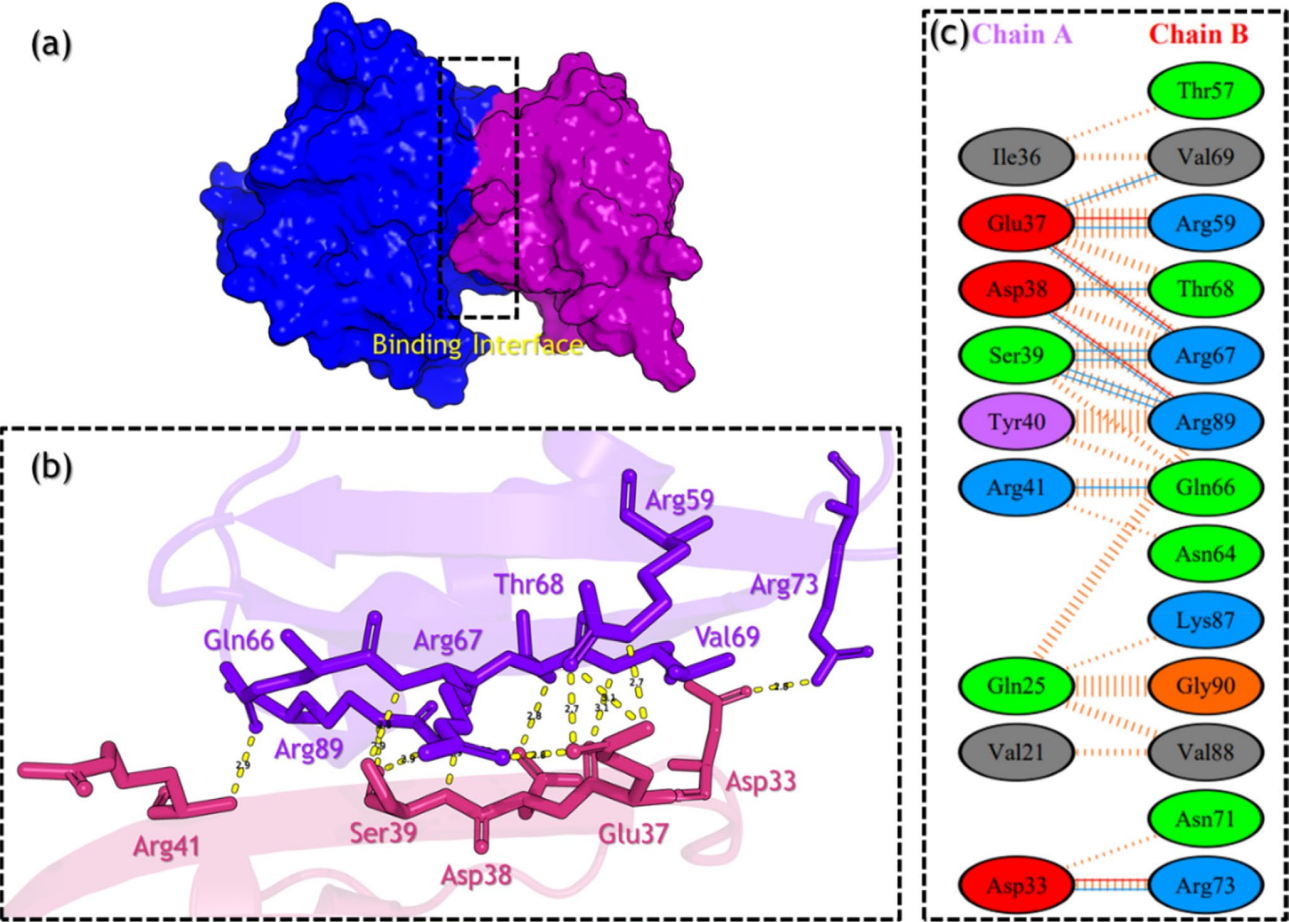
Interaction analysis of the wild‐type complex using the MD equilibrated structure. The part figure (a) shows the wild‐type complex binding, (b) shows the 3D interaction pattern for the wild‐type complex, and (c) shows the 2D interaction pattern for the wild‐type complex.

In the wild‐type, 11 hydrogen bonds between the two structures were reported which involves Asp33‐Arg73 (2.77 Å), Glu37‐Val69 (3.10 Å), Glu37‐Arg59 (2.72 Å), Glu37‐Arg67 (2.83 Å), Asp38‐Thr68 (2.83 Å), Asp38‐Arg89 (2.79 Å), Ser39‐Arg67 (2.89 Å), Ser39‐Arg89 (2.93 Å), Ser39‐Arg89 (2.88 Å), Ser39‐Arg67 (2.78 Å), and Arg41‐Gln66 (2.92 Å), while 4 salt‐bridges include Asp33‐Arg73 (2.77 Å), Glu37‐Arg59 (2.68 Å), Glu37‐Arg67 (2.83 Å), and Asp38‐Arg89 (2.79 Å). In addition, 90 non‐bonded contacts were also reported in the wild‐type complex. The interaction patterns of the wild‐type (RAF1–RAP1A) are shown in Figure 7a–c.

**FIGURE 8:**
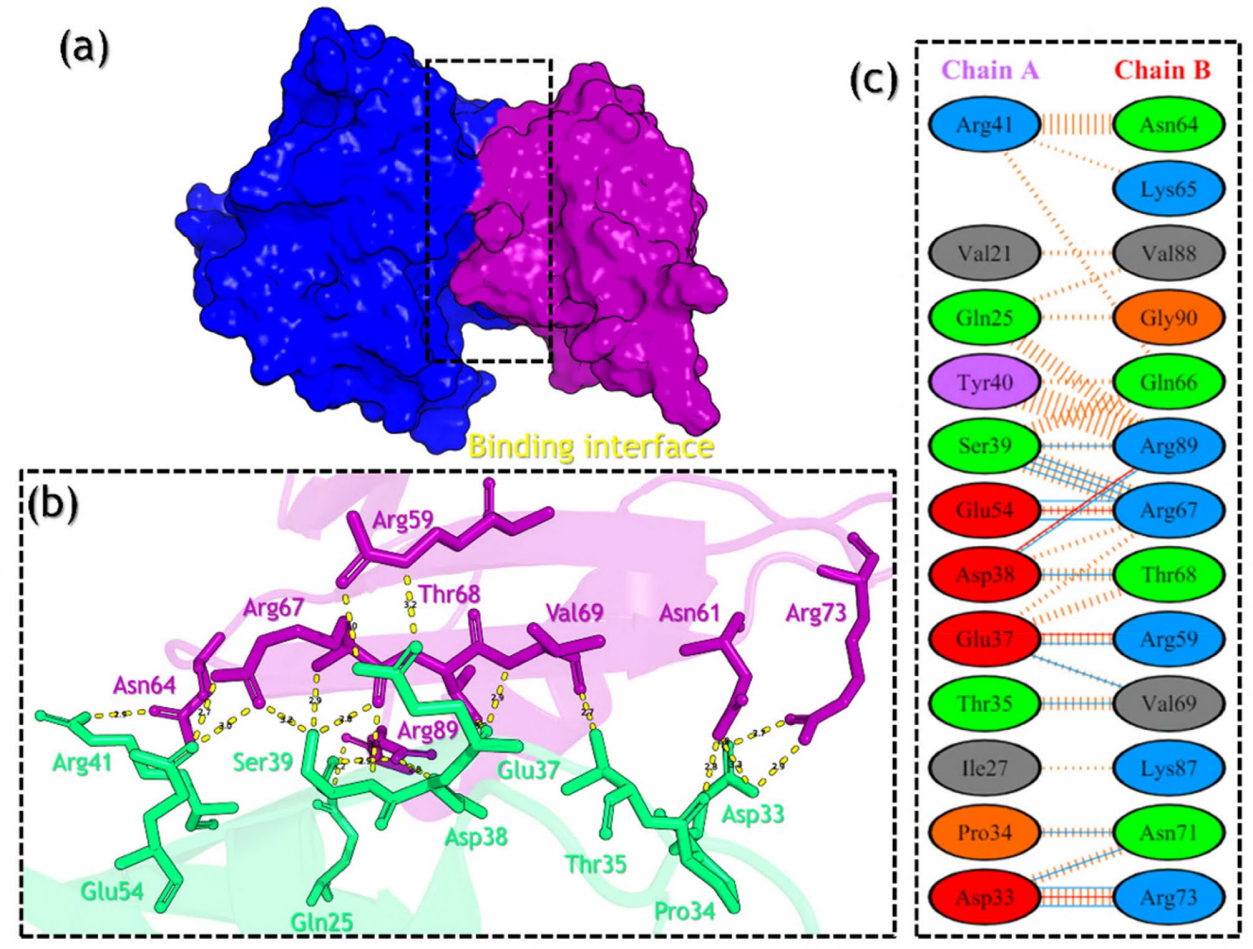
Interaction analysis of the V60F complex using the MD equilibrated structure. The part figure (a) shows the V60F complex binding, (b) shows the 3D interaction pattern for the V60F complex, and (c) shows the 2D interaction pattern for the V60F complex.

Furthermore, the V60F complex reported 15 hydrogen bonds including Asp33‐Arg73 (2.88 Å), Asp33‐Asn71 (2.82 Å), Asp33‐Arg73 (2.71 Å), Pro34‐Asn71 (2.84 Å), Thr35‐Val69 (2.69 Å), Glu37‐Val69 (2.91 Å), Glu37‐Arg59 (3.21 Å), Asp38‐Thr68 (2.86 Å), Asp38‐Arg89 (2.77 Å), Ser39‐Arg67 (3.15 Å), Ser39‐Arg89 (2.88 Å), Ser39‐Arg67 (2.85 Å), Ser39‐Arg67 (2.77 Å), Glu54‐Arg67 (3.02 Å), and Glu54‐Arg67 (2.73 Å), while four salt‐bridges which include Asp33‐Arg73 (2.71 Å), Glu37‐Arg59 (2.97 Å), Asp38‐Arg89 (2.77 Å), and Glu54‐Arg67 (2.73 Å). A total of 117 non‐bonded contacts were also reported in the V60F complex. The interaction patterns of the V60F mutant (RAF1–RAP1A) are shown in Figure 8a–c.

**FIGURE 9:**
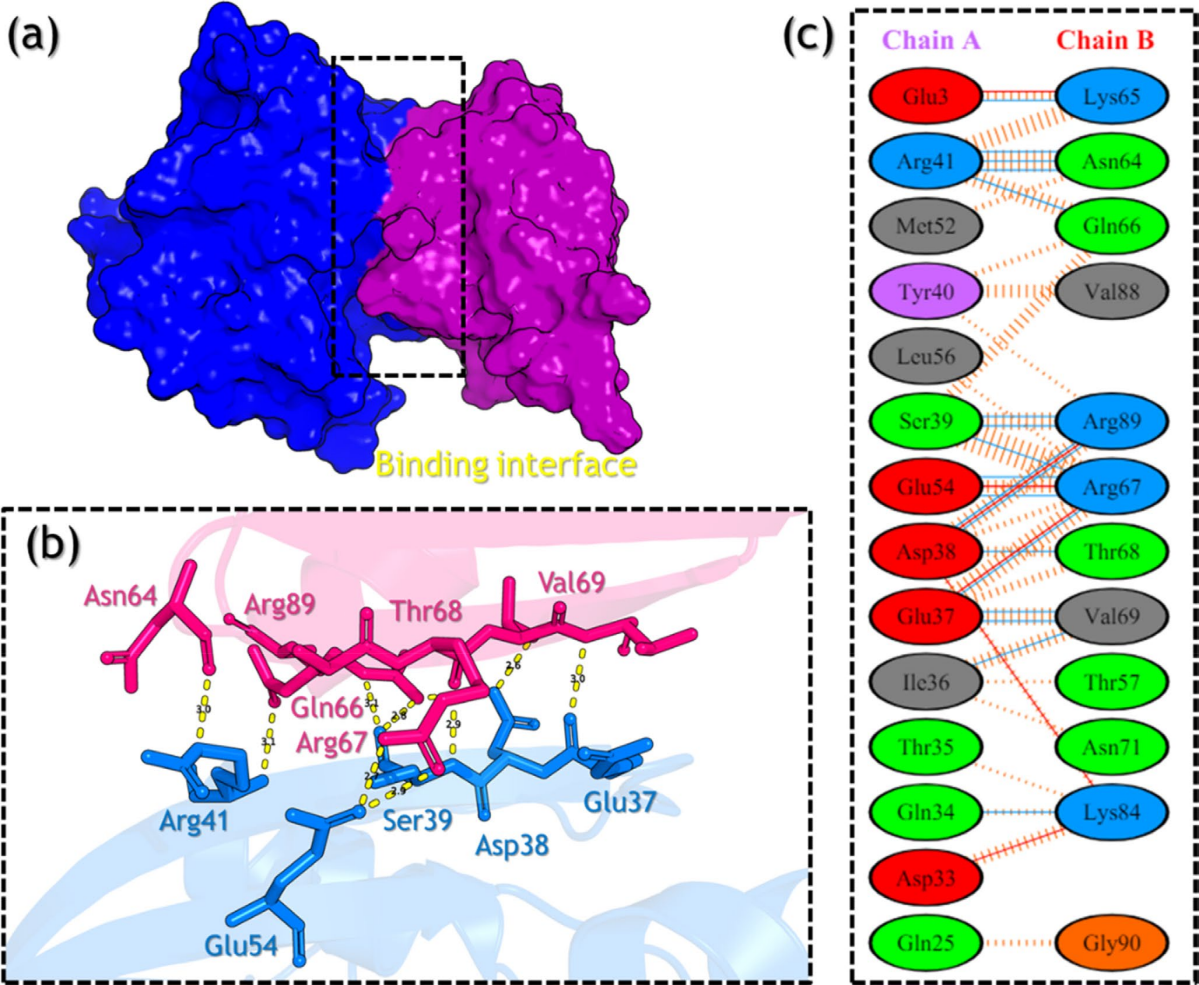
Interaction analysis of the P34Q complex using the MD equilibrated structure. The part figure (a) shows the P34Q complex binding, (b) shows the 3D interaction pattern for the P34Q complex, and (c) shows the 2D interaction pattern for the P34Q complex.

Moreover, the P34Q mutant reported 18 hydrogen bonds including Glu3‐Lys65 (2.70 Å), Gln34‐Lys84 (2.80 Å), Ile36‐Val69 (3.15 Å), Glu37‐Val69 (3.33 Å), Glu37‐Val69 (2.85 Å), Glu37‐Arg67 (2.75 Å), Asp38‐Thr68 (2.65 Å), Asp38‐Arg89 (2.75 Å), Asp38‐Arg89 (2.76 Å), Ser39‐Arg67 (2.86 Å), Ser39‐Arg89 (2.95 Å), Ser39‐Arg89 (2.78 Å), Arg41‐Gln66 (2.91 Å), Arg41‐Asn64 (2.70 Å), Arg41‐Asn64 (3.33 Å), Arg41‐Asn64 (3.01 Å), Glu54‐Arg67 (2.75 Å), and Glu54‐Arg67 (2.73 Å), while 6 salt‐bridges involving Glu3‐Lys65 (2.70 Å), Asp33‐Lys84 (2.72 Å), Glu37‐Arg67 (2.75 Å), Asp38‐Lys84 (3.89 Å), Asp38‐Arg89 (2.75 Å), and Glu54‐Arg67 (2.73 Å). In the P34Q complex, 119 non‐bonded contacts are reported. The interaction patterns for the P34Q are shown in Figure 9a–c. Based on the equilibrated structure of MD simulations, it is evident that the V60F and P34Q mutants exhibit increased binding between RAF1 and RAP1A compared with the wild‐type. Both mutants demonstrate a higher number of hydrogen bonds and salt bridges, indicating stronger intermolecular interactions. The mutants show the most pronounced enhancement in binding affinity, as they form additional hydrogen bonds and salt bridges, suggesting a potential for improved stability and functionality of the protein complex.

### 3.9 PCA of the Wild‐Type and Mutants

**FIGURE 10:**
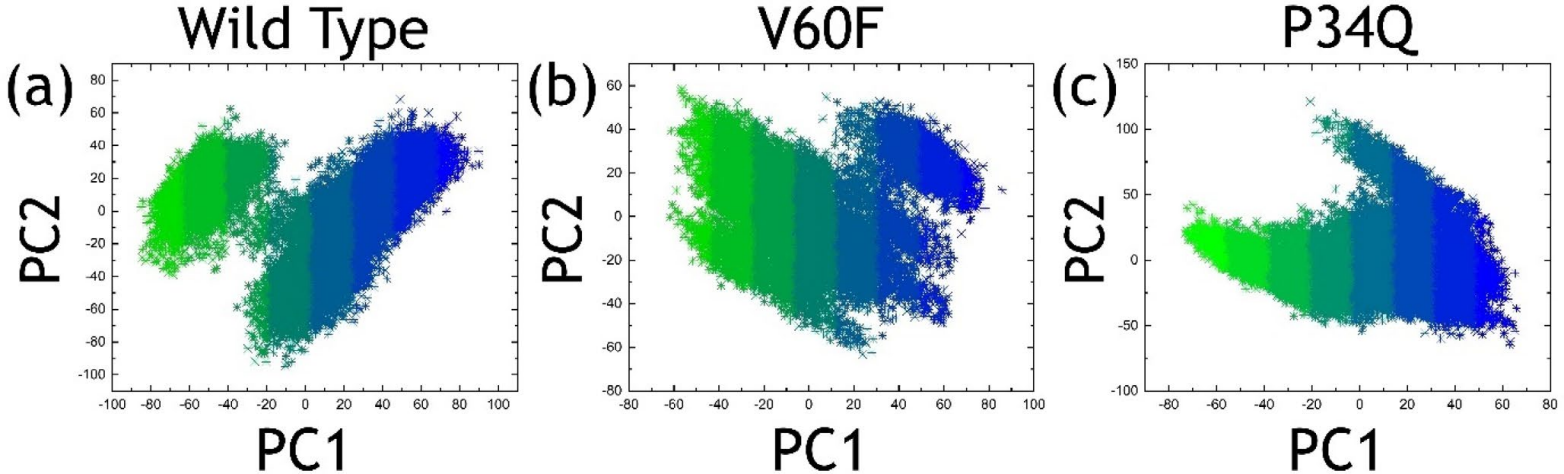
PCA graphs for the wild‐type and mutant complexes. The part figure (a) shows the PCA graph for the wild‐type complex, (b) shows the PCA graph for the V60F complex, and (c) shows the PCA graph for the P34Q complex. The blue color represents the one state while the mix blue and green represents the transition state and the green color represents another state.

PCA, a statistical method, can be used to understand the motions by dividing the components into uncorrelated variables known as principal components. These components are arranged in order of the amount of variance they capture, with the first component explaining the highest variance and subsequent components explaining decreasing amounts of variance. In molecular dynamics (MD) trajectories, PCA is commonly utilized to identify the major conformational motions, termed “essential dynamics,” of a protein. The fluctuation in conformation from frame to frame can be described as a linear combination of these essential dynamics identified by PCA. To determine the variations in the internal motion of each trajectory we also calculated PCA for the wild‐type and mutant complexes. The PCA graphs are depicted in Figure 10a–c. The PCA graphs reveal distinct conformational dynamics among the wild‐type and mutant complexes. The similarity in spreading along the *x* and *y* axes between the wild‐type and V60F mutant suggests an overall comparable conformational flexibility. However, the presence of two low‐energy conformational states in these complexes indicates a degree of structural heterogeneity. In contrast, the P34Q mutant with a larger spreading along both axes and a single energy conformational state suggests an increased conformational variability and a potential for a more diverse ensemble of structures. These findings underscore the impact of mutations on the protein's conformational landscape, highlighting their role in modulating structural dynamics and potentially influencing functional properties.

### 3.10 FEL Analysis

**FIGURE 11:**
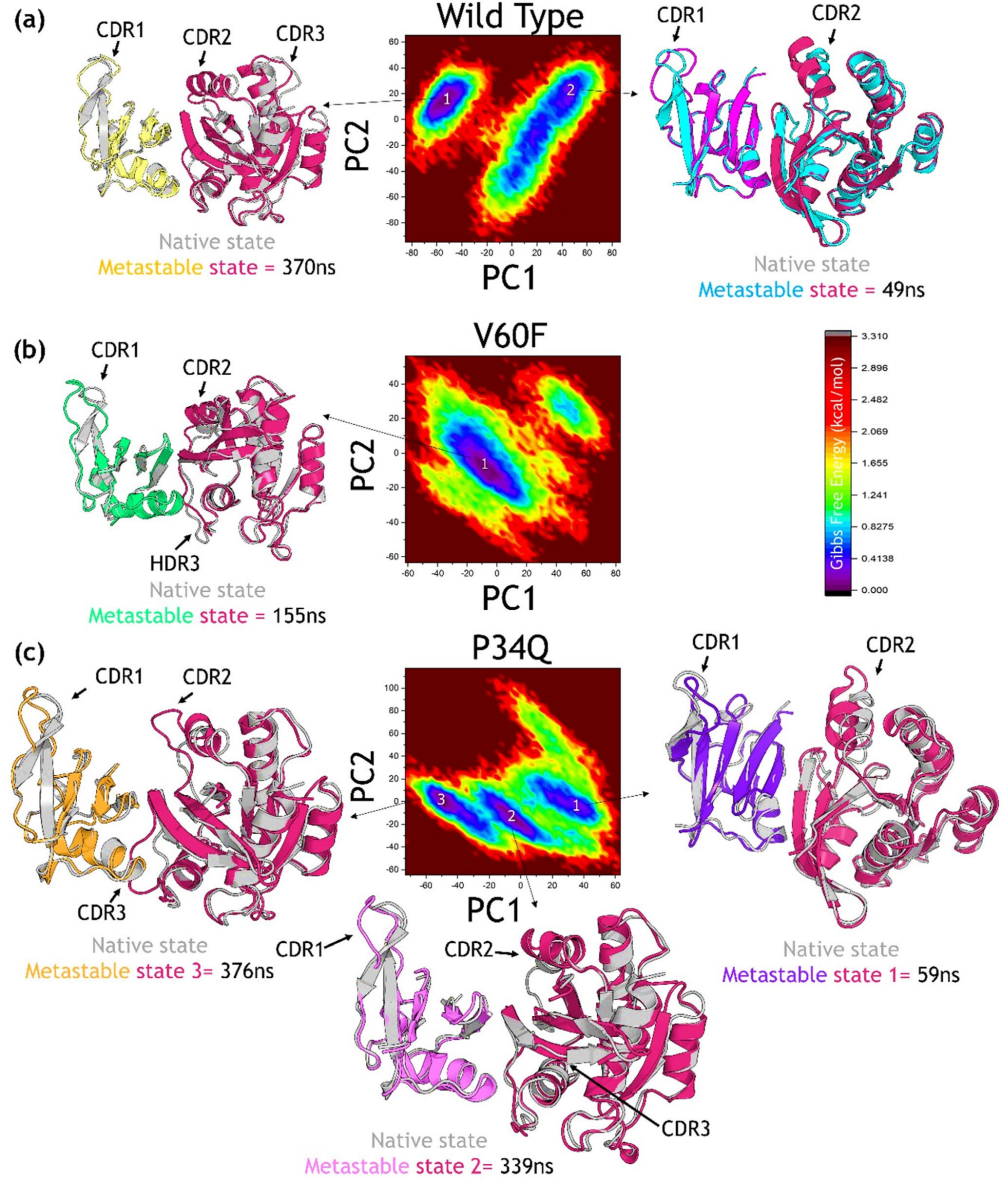
Free Energy Analysis (FEL) graphs for the wild‐type and mutant complexes. The part figure (a) shows the FEL graph for the wild‐type complex, (b) shows the FEL graph for the V60F complex, and (c) shows the FEL graph for the P34Q complex.

We used the two PCs to construct the FEL for each complex using the simulation trajectory. The wild‐type demonstrated two conformational states, P34Q three conformational states while the V60F reported one single energy state. The observation of two distinct conformational states in the wild‐type and three in the P34Q mutant, while the V60F mutant shows one single conformation, suggests intriguing dynamics influenced by the introduced mutations. Despite both mutants exhibiting lower BFE compared with the wild‐type, indicating stronger binding affinities, their differing conformational behavior hints at nuanced structural effects. The wild‐type and P34Q mutant may undergo conformational transitions between energetically favorable states, reflecting a dynamic equilibrium essential for biological function. We compared the native structure with the representative structures from the FEL graph basin and presented in Figure 11a. The wild type when compared with the representative structure reported two at 49 ns and three conformationally dynamic regions (CDR1‐3) at 376 ns. The first CDR1 region corresponds to 214–224 where it can be seen that the beta‐sheet has been converted to the loop which increases the flexibility of this region. Furthermore, the CDR2 corresponds to 63–72 where a proper helix has deviated from the native state and therefore causes an impact on the structure. Moreover, the CDR3 region which corresponds to 102–110 which is a loop region also determined significant deviation from the native state and therefore contributed to the differential dynamics of the wild type. In contrast, the V60F mutant displaying one single conformation implies a stabilized, possibly more rigid structure resulting from the mutation. This stabilization might facilitate enhanced binding by favoring a specific conformation conducive to stronger RAF1–RAP1A interactions. The metastable state also revealed three conformationally dynamic regions where the CDR1 and CDR2 are similar to the wild type however the region CDR3 which corresponds to 26–37 present at the interface also reported flexibility and therefore potentially showed enhanced activity of this mutation in the context of increased binding. The FEL graph along with the native and representative structures are given in Figure 11b. As mentioned above the P34Q demonstrated three metastable states so we retrieved the representative structures from the trajectory and compared with the native state. The FEL graph and structures are given in Figure 11b. The first metastable state at 59 ns reported only two CDR regions which correspond to the same positions as the wild type and V60F. The second metastable state reported at 339 ns also reported three CDR regions with the two regions CDR1 and CDR2 as the same as previous while the CDR3 here corresponds to 37–46 and a lengthy beta‐sheet can be seen to have split in two beta‐sheet connected by a newly adapted secondary structure, loop, during the simulation. Similarly, the third metastable state that occurred at 376 ns reported similar CDR1 and CDR2 variations however, the CDR3 region which corresponds to 191–202 amino acids has adapted a helix structure which is also present in the interface site and therefore the binding has affected. The FEL graph and the representative structures are given in Figure 11c. Thus, while all mutants exhibit improved binding, their distinct conformational dynamics underscore the complexity of molecular interactions influenced by these mutations, offering insights into structure–function relationships that are critical for protein engineering and drug design. The FEL graphs for the wild‐type and mutants are shown in Figure 11a–c.

### 3.11 BFE Calculation

The MM/GBSA technique, commonly utilized for calculating the BFE of biological partners, serves as a prevalent method for analyzing potential docking configurations. In comparison to alchemical free energy methods, this technique offers a less costly alternative while providing insights into the binding stability of crucial interaction regions and the BFE. Notably, it is considered more accurate than many rational scoring functions [23, 34, 40, 41]. Using the MM/GBSA technique enables us to explore the impact of mutations, that is, V60F and P34Q on the coupling of RAF1 and RAP1A. The wild‐type complex reported a Van der Waals (vdW) force value of −61.33 ± 0.28 kcal/mol, the V60F reported a vdW value of −66.60 ± 0.32 kcal/mol, while the P34Q complex reported a vdW value of −70.49 ± 0.90 kcal/mol. Moreover, the values of electrostatic energy were calculated to be −608.36 ± 2.90 kcal/mol for the wild‐type complex, −753.67 ± 2.72 kcal/mol for the V60F complex, and −706.35 ± 5.08 kcal/mol for the P34Q complex. Finally, the total BFE for each complex was estimated to be −71.94 ± 0.38 kcal/mol for the wild‐type, −95.57 ± 0.37 kcal/mol for the V60F, and −85.76 ± 0.72 kcal/mol for the P34Q complex. This clearly shows that these mutations do not only affect the conformational dynamics but also increase the binding of RAF1 and RAP1A which consequently activate the cancer pathways associated with these proteins. Upon analysis of the data, it is evident that each system demonstrates a rise in free energy in the gas phase while remaining unchanged in the solvent state. These findings suggest that the thermodynamic preference for these proteins binding is predominantly influenced by enthalpic factors, with favorable interactions prevailing in the gas phase. Conversely, binding is deemed unfavorable in terms of entropy due to the detrimental impact of solvation. These findings underscore the role of these mutations on the coupling and RAF1–RAP1A and therefore can be used as a starting point for the design and development of potential inhibitors. The BFE results are summarized in Table 5.

**TABLE 5:** BFE results are calculated through the MM‐GBSA approach (all the energies are provided in kcal/mol).

Parameters	MM‐GBSA		
	WT	V60F	P34Q
VDWAALS	−61.33 ± 0.28	−66.60 ± 0.32	−70.49 ± 0.90
EEL	−608.36 ± 2.90	−753.67 ± 2.72	−706.35 ± 5.08
EGB	605.90 ± 2.80	734.62 ± 2.49	699.53 ± 5.04
ESURF	−8.14 ± 0.03	−9.91 ± 0.02	−8.45 ± 0.05
DELTA G Gas	−733.75 ± 2.87	−811.28 ± 2.65	−642.84 ± 5.10
DELTA G Solv	676.69 ± 2.79	734.70 ± 2.49	596.08 ± 5.03
DELTA G	−71.94 ± 0.38	−95.57 ± 0.37	−85.76 ± 0.72

## 4 Conclusions

The current study underscores the critical role of RAF1–RAP1A interaction in activating the MAPK/ERK pathway, which is pivotal in cancer progression. Understanding aberrant interactions driven by clinical mutations is essential for targeted therapies. Through predictive algorithms and molecular simulations, we identified deleterious mutations in RAF1 and RAP1A, shedding light on their impact on cancer patient survival. Notably, mutations altering the binding affinity between RAF1 and RAP1A were observed, indicating their potential as therapeutic targets. Protein–protein docking and molecular simulations revealed structural and dynamic alterations in mutant complexes, highlighting their significance in cancer pathogenesis. These findings emphasize the importance of targeting RAF1–RAP1A interaction for cancer therapy, particularly through the modulation of the activity of the MAPK/ERK signaling pathway.

## Author Contributions

**Abbas Khan:** conceptualization, investigation, funding acquisition, writing – original draft, software, validation, formal analysis, writing – review and editing, data curation. **Syed Shujait Ali:** conceptualization, methodology, validation, visualization, formal analysis, data curation. **Muhammad Ammar Zahid:** conceptualization, visualization, validation, methodology, data curation. **Shahenda Salah Abdelsalam:** conceptualization, validation, writing – review and editing, visualization. **Noorah Albekairi:** conceptualization, methodology, software, data curation, resources, project administration, visualization, funding acquisition. **Raed M. Al‐Zoubi:** funding acquisition, conceptualization, validation, visualization, project administration, formal analysis, data curation. **Mohanad Shkoor:** conceptualization, methodology, visualization, funding acquisition, project administration, formal analysis, resources. **Dong‐Qing Wei:** conceptualization, investigation, visualization, validation, methodology, writing – original draft, writing – review and editing, supervision, resources. **Abdelali Agouni:** conceptualization, methodology, validation, visualization, funding acquisition, writing – original draft, project administration, writing – review and editing, software, supervision, resources.

## Ethics Statement

The authors have nothing to report.

## Consent

All authors approved the manuscript.

## Conflicts of Interest

The authors declare no conflicts of interest.

### Peer Review

The peer review history for this article is available at https://www.webofscience.com/api/gateway/wos/peer‐review/10.1002/prot.26759.

## Data Availability

All the data is available on RCSB, UniProt and any simulation data would be provided on demand. The accession numbers to access this data are given in the manuscript.
